# The cell-end protein Tea4 spatially regulates hyphal branch initiation and appressorium remodeling in the blast fungus *Magnaporthe oryzae*

**DOI:** 10.1091/mbc.E23-06-0214

**Published:** 2023-12-14

**Authors:** Audra Mae Rogers, Rachel Taylor, Martin John Egan

**Affiliations:** a Department of Entomology and Plant Pathology, University of Arkansas Systems Division of Agriculture, Fayetteville, AR 72701; University of North Carolina, Chapel Hill

## Abstract

The differentiation of specialized infection cells, called appressoria, from polarized germ tubes of the blast fungus *Magnaporthe oryzae*, requires remarkable remodeling of cell polarity and architecture, yet our understanding of this process remains incomplete. Here we investigate the behavior and role of cell-end marker proteins in appressorium remodeling and hyphal branch emergence. We show that the SH3 domain-containing protein Tea4 is required for the normal formation of an F-actin ring at Tea1-GFP-labeled polarity nodes, which contributes to the remodeling of septin structures and repolarization of the appressorium. Further, we show that Tea1 localizes to a cortical structure during hyphal septation which, unlike contractile septin rings, persists after septum formation, and, in combination with other polarity determinants, likely spatially regulates branch emergence. Genetic loss of Tea4 leads to mislocalization of Tea1 at the hyphal apex and with it, impaired growth directionality. In contrast, Tea1 is largely depleted from septation events in Δ*tea4* mutants and branching and septation are significantly reduced. Together, our data provide new insight into polarity remodeling during infection-related and vegetative growth by the blast fungus.

## Abstract (executive summary)

The fungus Magnaporthe oryzae differentiates a specialized infection cell called an appressorium to infect host plants. Appressorium functionality requires the actin-mediated remodeling of a septin disk into a ring within its base, but precisely how this process is orchestrated is not well understood.

The authors use a quantitative live-cell imaging and reverse-genetic approach to investigate the importance of Tea cell-end marker proteins for appressorium repolarization and hyphal growth.

They found that Tea4 is required for normal appressorium remodeling and hyphal branching. Improved mechanistic understanding of appressorium development and hyphal branching may inform novel control strategies to tackle fungal pathogens.

## INTRODUCTION

**FIGURE 1:**
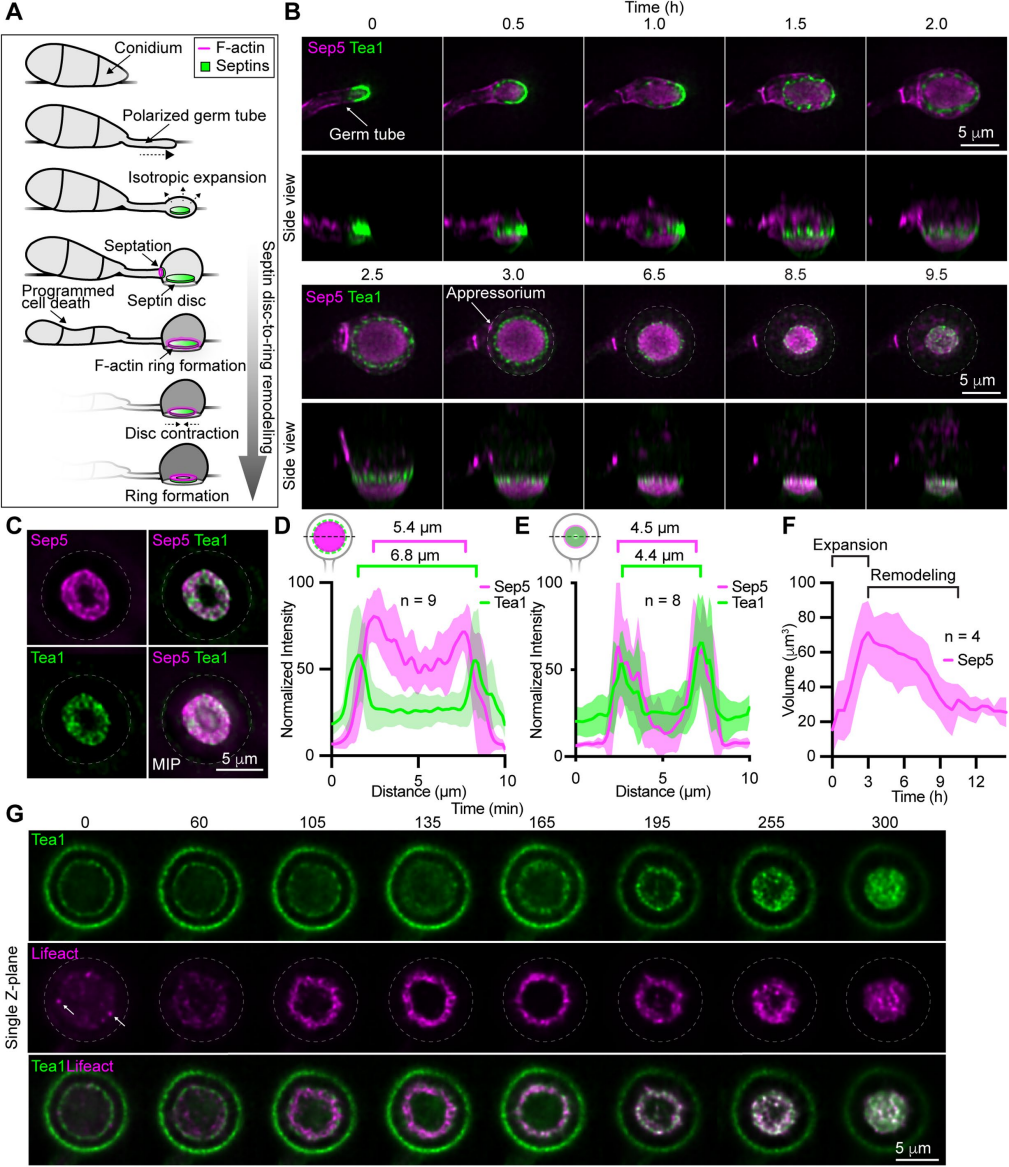
Tea1 localizes to cortical clusters around septin disks before their remodeling into rings. (A) Cartoon depicting relevant morphogenetic events during appressorium development. (B) Time series showing the localization of Tea1-GFP and Sep5-RFP in a developing appressorium. Images represent maximum intensity projections of Z-series. (C) Fluorescence micrographs showing Sep5-RFP and Tea1-GFP localization in an appressorium 16 h post inoculation (hpi). MIP = maximum intensity projection. (D and E) Fluorescence intensity distribution plots generated from line scans through appressoria, as depicted in cartoons, at 3 hpi (D) and 9 hpi (E). Solid lines connect mean values and shaded areas represent ± s.d. (E and F) Plot showing the mean volume (solid line) ± s.d. (shaded areas) of three-dimensional rendered Sep5-RFP-labeled septin structures within developing appressoria. (G) Time series showing the localization of Tea1-GFP (upper panels) and Lifeact-RFP (middle panels) in the base of a maturing appressorium. Dashed lines outline the appressorium and white arrows highlight putative F-actin nucleation events.

Filamentous fungi produce extremely polarized cylindrical cells called hyphae, which grow indefinitely via the cytoskeleton-mediated delivery of secretory vesicles to the expanding apex (Steinberg *et al.*, 2017). The establishment and maintenance of polarity in filamentous fungi, and fission yeast, involves the microtubule-dependent delivery of cell-end marker proteins, including the Tea (for *tip elongation aberrant*) proteins, to the cell cortex (Fischer *et al.*, 2008; Chang and Martin, 2009). Here, one of their functions is to recruit actin nucleating proteins to establish F-actin arrays which support polarized exocytosis (Martin *et al.*, 2005; Higashitsuji *et al.*, 2009). Polarized hyphal cells can undergo cytokinesis, culminating in the deposition of cross walls, called septa, which serve to compartmentalize hyphae. Critical to the success of filamentous fungi, which occupy diverse ecological niches, and include pathogens of plant, insects and humans, is their ability to form interconnected branched networks of hyphae. Branching is spatially regulated, and lateral branch sites, in some fungi, emerge adjacent to existing septa (Harris, 2008). How these sites are selected, and new axis of polarity established, remains unclear, as does the involvement of Tea proteins in this process. Importantly, filamentous fungi also differentiate morphologically diverse structures from polarized hyphae, such as spores, fruiting bodies, and specialized infection apparatus. The blast fungus, *Maganporthe oryzae*, which destroys rice crops, forms a pressurized infection cell called an appressorium to rupture the leaf cuticle (Ryder *et al.*, 2022). The appressorium differentiates from the apex of a narrow germ tube, which, under conducive conditions, undergoes a switch from polarized to isotropic growth (Figure 1A). Precisely how this switch is achieved mechanistically remains unclear. As the appressorium expands, a cortical septin disk forms within its base which supports a vertical array of microtubules, orientated with their plus-ends pointing towards the septin disk (Dulal *et al.*, 2020, 2021). Around the time the appressorium reaches full size, a septum is deposited in the germ tube neck, partitioning the infection cell from the conidium, which undergoes programmed autophagic cell death (Figure 1A; Veneault-Fourrey *et al.*, 2006). Soon after, and in response to the appressorium reaching a critical turgor threshold (Ryder *et al.*, 2019), the septin disk is remodeled into a toroidal ring (Dagdas *et al.*, 2012), in a dynamic process involving the formation and constriction of an F-actin ring (Figure 1A; Dulal *et al.*, 2021). These events facilitate the repolarization of the appressorium, and enable the formation of a polarized penetration hypha from its base (Howard *et al.*, 1991). How these infection-specific polarity and cytoskeleton remodeling events are coordinated in space and time remains largely unknown. Here, we investigate the behavior and role of *M. oryzae* orthologues of the Tea proteins, Tea1 and Tea4, in appressorium remodeling and the spatial control of hyphal branch emergence.

## RESULTS AND DISCUSSION

To gain new insight into the behavior of polarity determinants during the transition from polarized to isotropic growth that occurs during the differentiation of appressoria, we generated a strain in which the cell-end marker and kelch-repeat protein, Tea1, was tagged with GFP (Dagdas *et al.*, 2012), and the septin, Sep5, was tagged with RFP (Dulal *et al.*, 2021), and imaged appressorium differentiation in vitro (Rogers *et al.*, 2021). Consistent with its known role as a cell-end marker (Qu *et al.*, 2022), Tea1-GFP localized to the leading edge of apically expanding germ tubes and was enriched at the equatorial plane (Figure 1B). The transition from polarized to isotropic expansion saw the redistribution of Tea1-GFP from the leading edge of the germ tube, to a ring of discrete nodes peripheral to cortical septin disks (Figure 1B). Interestingly, components of the exocyst complex, which mediates the tethering of post-Golgi secretory vesicles to the plasma membrane for polarized exocytosis, localize in a similar ring confirmation (Gupta *et al.*, 2015). As appressorium differentiation proceeds, cortical septin disks, like the appressorium itself, undergo a period of expansion, before contracting and being remodeled into rings (Figure 1, B–F; Dulal *et al.*, 2021). Following the remodeling of the septin disk, Tea1-GFP redistributed from peripheral nodes to colocalize with the Sep5-RFP-labeled ring-like structure (Figure 1, B–E). The contraction of septin disks occurs following the emergence of an F-actin ring at their periphery, which promotes this remodeling process (Dulal *et al.*, 2021). In other fungi, cell-end proteins are involved in the recruitment of formins, and other actin nucleation and elongation factors (Martin *et al.*, 2005; Higashitsuji *et al.*, 2009). We, therefore, hypothesized that the appressorium F-actin ring might be nucleated from Tea1 nodes before septin disk-to-ring remodeling. To test this idea, we generated a strain coexpressing Tea1-GFP and Lifeact-RFP and imaged the formation of the F-actin ring at the base of the appressorium relative to the localization of Tea1-GFP (Figure 1G). Before the formation of the F-actin ring, discrete Lifeact-RFP-labeled puncta, which we speculate represent nucleation sites, colocalized with the Tea1-GFP-labeled nodes (Figure 1G). As F-actin emerged and organized into a ring, these appeared to only partially colocalize with Tea1-GFP-labeled nodes. Later, as the ring began to constrict, colocalization between the Tea1-GFP-labeled nodal ring and Lifeact-RFP-labeled F-actin was again more evident (Figure 1G). Thus, the redistribution of Tea1-containing polarity nodes to the periphery of cortical septin disks precedes septin disk-to-ring remodeling, and these nodes spatiotemporally colocalize with sites of F-actin nucleation.

**FIGURE 2:**
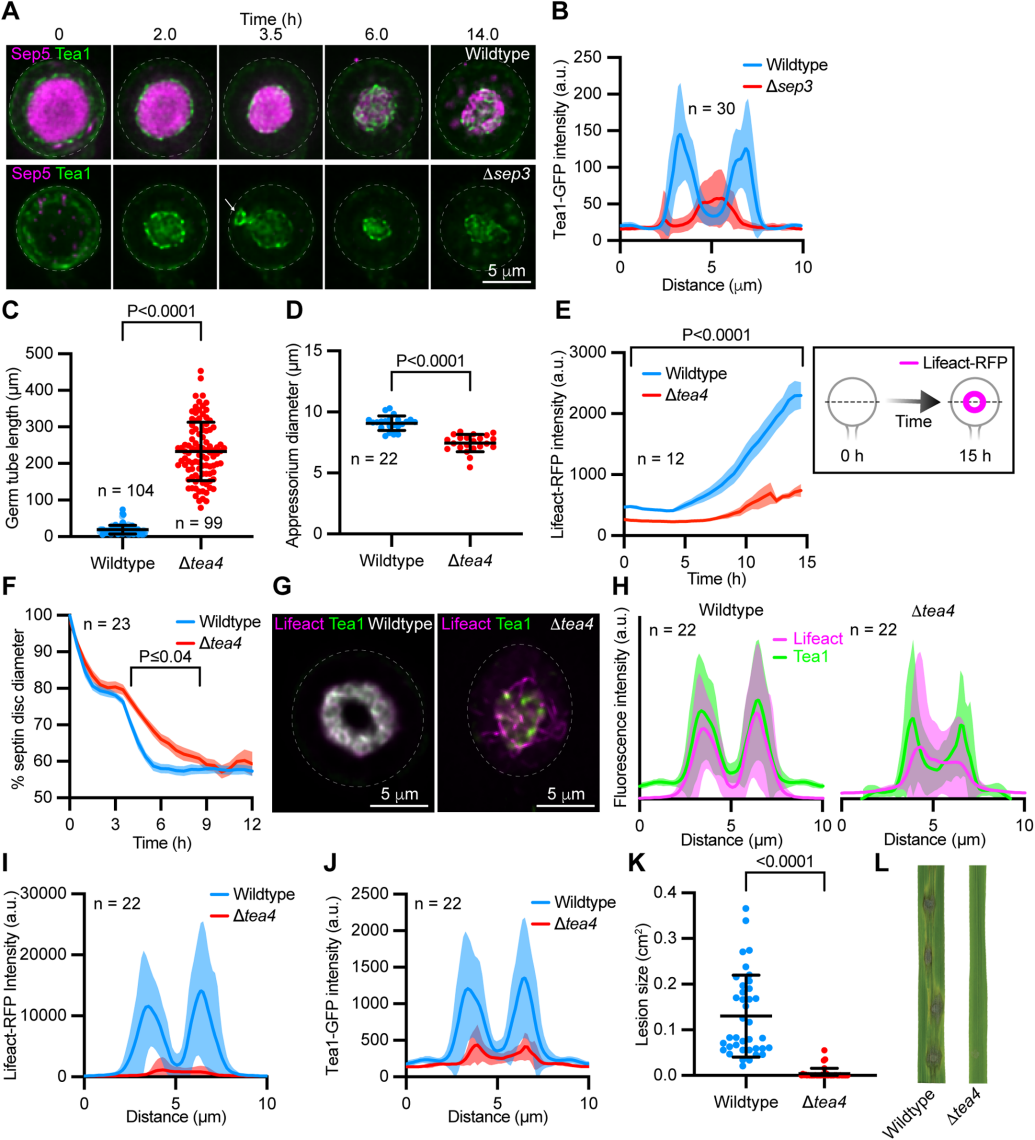
Appressorium morphogenesis is perturbed in the genetic absence of Tea4. (A) Time series showing the localization of Tea1-GFP and Sep5-RFP in developing appressoria from the wild-type (upper panels) and a Δ*sep3* mutant (lower panels). Images represent maximum intensity projections of Z-series. White arrow indicates aberrant cortical Tea1 patches. (B) Tea1-GFP fluorescence intensity distribution plots generated from line scans through appressoria of the wild-type and Δ*sep3* mutant 16 hpi. Solid lines connect mean values and shaded areas represent ± s.d. (C and D) Plot showing mean germ tube length (C) and appressorium diameter (D) in wild-type and Δ*tea4* mutants ± s.d. (E) Plot showing the mean (solid line) ± s.e.m (shaded regions) fluorescence intensity of Lifeact-RFP within wild-type and Δ*tea4* mutant appressoria over time and a schematic outlining the analysis (box). (F) Plot showing the normalized diameter of Sep5-GFP-labeled septin structures in the appressoria of the wild-type strain versus Δ*tea4* mutants over time. (G and H) Fluorescence images showing Tea1-GFP and Lifeact-RFP localization in a wild-type (left panel) and Δ*tea4* (right panel) appressorium (G) and corresponding normalized fluorescence intensity distribution plots generated from line scans through wild-type (left plot) and Δ*tea4* (right plot) appressoria 48 hpi (H) Brightness and contrast of representative images are not scaled equally. (I and J) Plots generated from line scans through wild-type and Δ*tea4* appressoria comparing Lifeact-RFP (I) and Tea1-GFP (J) fluorescence intensity distribution. K and L) Plot showing the mean ± s.d. blast lesion size on susceptible rice leaves inoculated with wild-type and Δ*tea4* conidia (K) and representative images showing blast disease lesions on rice leaves (L). *P* values report statistical significance as determined by unpaired *t* test (C, D, and K), or two-way mixed model ANOVA with corrections for multiple comparisons using two-stage step up method of Benjamini, Krieger, and Yekutieli (*q* = 0.05; E and F).

We sought to determine the functional relationship between the septin cytoskeleton and Tea1-GFP-labeled polarity nodes during appressorium morphogenesis. Septin structures act as diffusion barriers to constrain the lateral movement of membrane-associated proteins (Takizawa *et al.*, 2000), so we were curious as to whether appressorium septin disks were required to maintain the ring-like organization of Tea1-GFP, before disk-to-ring remodeling. We deleted the gene encoding for the core septin, Sep3, in a strain coexpressing Sep5-RFP and Tea1-GFP and imaged appressorium development in this mutant. Interestingly, while Tea1-GFP retained a nodal ring-like confirmation in the absence of a septin disk, Tea1-GFP also mislocalized to aberrant patches on the appressorium cortex (Figure 2A). Following the remodeling of septin disks into rings within wild-type appressoria, Tea1-GFP becomes tightly interwoven within the septin structure (Figures 1C and 2A). However, in Δ*sep3* mutants, which can’t form higher-order septin structures (Dagdas *et al.*, 2012), Tea1-GFP mislocalized within the base of the appressorium (Figure 2, A and B), as reported previously (Dagdas *et al.*, 2012). Thus, cortical septin structures help to spatially constrain the localization of polarity nodes during appressorium morphogenesis.

The cell-end protein, Tea4, has previously been shown to be important for normal appressorium differentiation and functionality by *M. oryzae* (Patkar *et al.*, 2010). In light of more recent findings (Dulal *et al.*, 2021), we sought to gain new insight into the role of this SH3 domain-containing protein in appressorium morphogenesis. In particular, given the importance of Tea4 for formin-mediated actin nucleation and extension in other fungi (Martin *et al.*, 2005; Higashitsuji *et al.*, 2009), we wondered how genetic loss of the *M. oryzae* Tea4 orthologue might impact appressorium repolarization and remodeling. We, therefore, deleted *TEA4* in various fluorescently labeled strains and observed infection-related development in Δ*tea4* mutants. Consistent with previous studies of both Δ*tea4* (Patkar *et al.*, 2010), and Δ*tea1* mutants in *M. oryzae* (Qu *et al.*, 2022), Δ*tea4* conidia produced extremely long germ tubes (Figure 2C). When appressoria did eventually form in Δ*tea4* mutants, they were smaller in size than the wild-type (Figure 2D). Interestingly, Lifeact-RFP-labeled F-actin failed to accumulate within the appressoria of Δ*tea4* mutants to the same extent that it did in wild-type cells during their maturation (Figure 2E), and furthermore, did not localize to conspicuous contractile F-actin rings (Supplemental Movie S1; Dulal *et al.*, 2021). Surprisingly, despite this fact, septin disks still underwent remodeling during appressorium maturation; however, the temporal dynamics of this process were significantly altered in Δ*tea4* mutants (Figure 2F). Following the maturation of wild-type appressoria, Lifeact-RFP-labeled F-actin decorated Tea1-GFP-labeled cortical rings and appeared tightly interwoven with these structures (Figure 2G, left panel), resulting in similar fluorescence distribution profiles (Figure 2H). In contrast, in Δ*tea4* mutant appressoria, F-actin was more loosely associated with Tea1-GFP-labeled structures (Figure 2G, right panel) as demonstrated by comparatively mismatched Tea1-GFP and Lifeact-RFP fluorescence intensity distribution profiles (Figure 2H). Compared with wild-type cells, mature Δ*tea4* mutant appressoria contained less Lifeact-RFP-labeled F-actin (Figure 2I) and Tea1-GFP was also less abundant in cortical ring-like structures (Figure 2J). Consistent with previous findings (Patkar *et al.*, 2010), Δ*tea4* mutants were largely unable to cause disease on a blast susceptible rice cultivar (Figure 2, K and L), likely due to the perturbed cortical remodeling and subcellular organization of their appressoria (Figure 2G). Thus, Tea4 is required for the normal formation of F-actin rings at polarity nodes which influence septin disk-to-ring remodeling and appressorium morphogenesis.

**Figure:** Movie S1 Time lapse sequence showing the localization of Tea1‐GFP and Lifeact‐RFP during appressorium morphogenesis in a Δ*tea4* mutant. Time is shown in hours. Movie plays at 5 fps.

**FIGURE 3:**
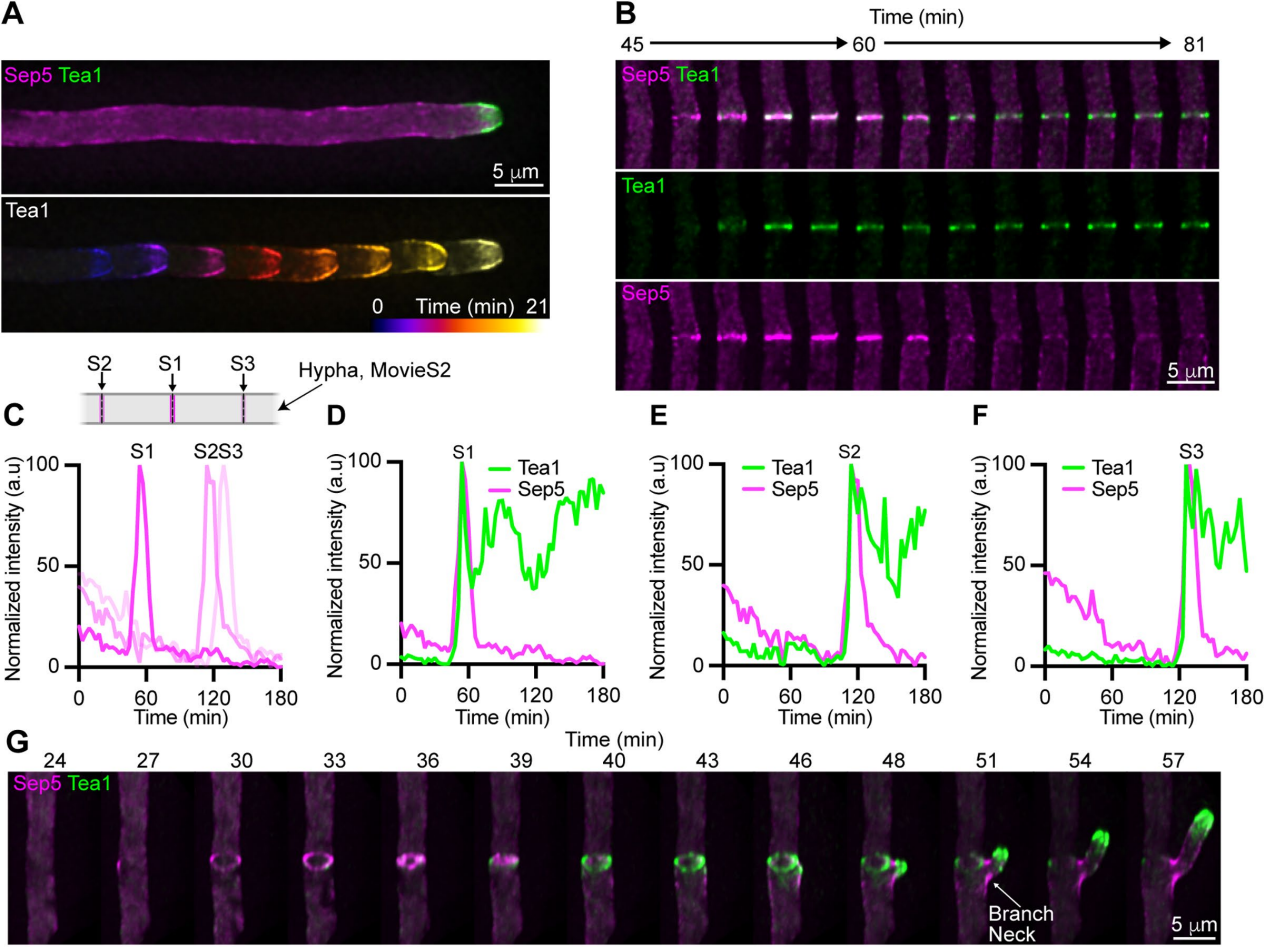
Tea1 localizes to cortical rings at septation sites. (A) Fluorescence image showing the localization of Tea1-GFP in a hypha (upper panel) and temporally color-coded projections of Tea1-GFP localization during polarized growth (lower panel). (B) Time-series showing the localization of Sep5-RFP (top and bottom panels) and Tea1-GFP (top and middle panels) during septation. Images represent maximum intensity projections of Z-series. (C–F) Plots showing the normalized intensity of Sep5-RFP (C) and Tea1-GFP (D–F) fluorescence at three septation events (Supplemental Figure S1–S3) occurring sequentially within a hypha over a period of 3 h, as depicted in schematic inset. (G) Time series showing the persistence of cortical Tea1-GFP-labeled rings following septin ring constriction and branch emergence. Images represent rotated maximum intensity projections.

We were curious as to the behavior of Tea1-GFP in hyphal cells, which extend indefinitely through apical expansion, but also undergo coordinated lateral branching events in which new polarity sites are established. Precisely how branching is spatially regulated remains unresolved, but the septin and F-actin cytoskeletons are thought to play fundamental roles in this process (Harris, 2019). As previously reported, Tea1-GFP localized to the growing hyphal tip of hyphal cells (Qu *et al.*, 2022), and appeared enriched in apical membranes (Figure 3A). Interestingly, Tea1-GFP, like Sep5-RFP, also localized to septation events (Figure 3B). However, unlike Sep5-RFP, which rapidly delocalized from septation sites, presumably following the completion of cytokinesis, Tea1-GFP persisted indefinitely at newly formed septa (Figure 3, A, D–F; Supplemental Movie S2). In *Aspergillus nidulans*, the Tea4 and Mod5 orthologues, TeaC and TeaR, respectively, both localize to septa, but TeaA, is restricted to the hyphal apex (Takeshita *et al.*, 2008; Higashitsuji *et al.*, 2009). Strikingly, four-dimensional fluorescence imaging of septation and branch emergence revealed that, while Sep5-RFP localized to cortical rings that underwent constriction within ∼10 min, Tea1-GFP-localized to noncontractile rings that remained at the cortex longer (Figure 3G; Supplemental Movie S3). Before branching, both Sep5-RFP and Tea1-GFP localized to the cortex, immediately below the newly formed septa, at branch emergence sites (Figure 3G ∼43 min). Interestingly, septins are thought to play a role in the repression of lateral branch emergence in fungi, as deletion of septin-encoding genes results in hyperbranching in various fungi (Lindsey *et al.*, 2010; Hernández-Rodríguez *et al.*, 2012; Berepiki and Read, 2013). While Sep5-RFP remained at sites of membrane curvature at the branch neck, Tea1-GFP localized to the new hyphal apex (Figure 3G). Unlike in nonbranch associated septa, where cortical Tea1-GFP persists indefinitely (Figure 3, B–F), Tea1-GFP was depleted from cortical rings upon branching (Figure 3G). Thus, Tea1’s retention at the septal cortex may serve to “seed” the formation of new polarity sites below existing septa, presumably following their activation by other factors, to control lateral branch emergence.

**Figure:** Movie S2 Time lapse sequence showing the dynamics of Tea1‐GFP and Sep5‐RFP during the sequential formation of three discrete septa within a single *M. oryzae* hypha, as quantified in Figure 2 (G‐E). Time is shown in hours. Movie plays at 5 fps.

**Figure:** Movie S3 Time lapse sequence showing the organization and dynamics of Tea1‐GFP, Sep5‐RFP, and calcofluor white‐labelled (CW) cell walls, during septation and branch emergence in a *M. oryzae* hypha. Time is shown in hours. Movie plays at 5 fps.

**FIGURE 4:**
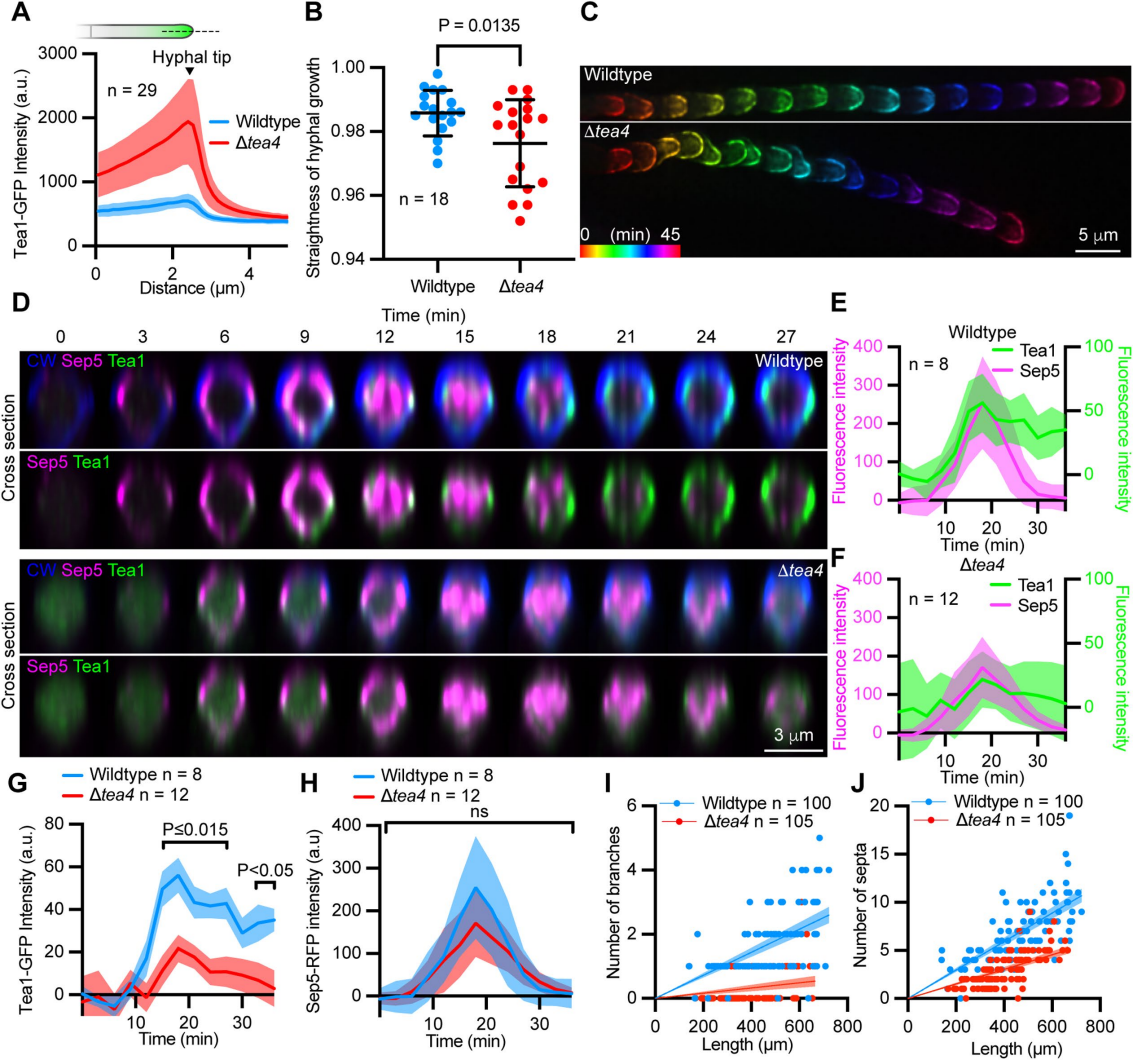
Tea1 is depleted from hyphal septation events in the genetic absence of Tea4. (A) Fluorescence intensity distribution plots generated from line scans through the tip of wild-type and Δ*tea4* hyphae, as depicted in schematic. Solid lines connect mean values and shaded areas represent ± s.d. (B) Plot showing the straightness of hyphal growth in wild-type and Δ*tea4* mutants. Mean ± s.e.m indicated. (C) Temporally color-coded projections of Tea1-GFP localization during polarized growth in wild-type (upper panel) and Δ*tea4* (lower panel). (D) Time series showing the localization of Sep5-RFP, Tea1-GFP, and calcofluor white (CW)-labeled cell wall during a septation event in a wild-type (upper panels) and Δ*tea4* (lower panels) hypha. CW fluorescence (blue) is omitted in respective lower panels for clarity. (E and F) Plots showing the intensity of Sep5-RFP and Tea1-GFP fluorescence at septation events in wild-type (E) and Δ*tea4* (F) over time. Shaded areas represent ± s.d. (G and H) Plots showing the intensity of Tea1-GFP (G) and Sep5-RFP (H) fluorescence in wild-type versus Δ*tea4* over time. Shaded areas represent ± s.e.m. in (G) and ± s.d. in (H). (I and J) Plots showing the number of branches (I) and septa (J) in wild-type and Δ*tea4* hyphae. Least squares regression analysis, sum-of-squares F test, constrained to X = 0 and Y = 0, 95% confidence interval mapped. *P* values report statistical significance as determined by unpaired *t* test (B), or two-way mixed model ANOVA with corrections for multiple comparisons using two-stage step up method of Benjamini, Krieger, and Yekutieli (*q* = 0.05; G and H).

Loss of Tea4 led to an increase in the amount of Tea1-GFP within hyphae (Figure 4A), and Δ*tea4* mutants extended in less straight trajectories than wild-type (Figure 4, B and C), however, their growth rates were not significantly different (wild type = 1.17 ± 0.4 μm/min [*n* = 18] vs. Δ*tea4* = 1.22 ± 0.4 μm/min [*n* = 19], *P* = 0.698, *t* test). Similar defects in the maintenance of growth directionality are well-reported for cell-end marker mutants in the *A. nidulans* (Takeshita *et al.*, 2008; Higashitsuji *et al.*, 2009). Strikingly, despite increased amounts of Tea1-GFP within the cytoplasm of Δ*tea4* mutant hyphae (Figure 4A), Tea1-GFP was either significantly depleted or entirely absent from septation events in the genetic absence of Tea4 (Figure 4, D–G). Furthermore, when Tea1-GFP could be detected at forming septa, it did not persist following the completion of septation to the same extent as it did in wild-type (Figure 4, D–G). In contrast, there was no significant difference in the amount of Sep5-RFP at any point during septation between wild-type and Δ*tea4* mutants (Figure 4H). In addition, cortical Sep5-RFP-labeled rings formed and seemed to contract normally in Δ*tea4* mutants despite the absence or depletion of Tea1-GFP (Figure 4D). We wondered whether loss of *TEA4*, and the corresponding depletion of Tea1-GFP following septation, might impact branch emergence in Δ*tea4* mutants. We, therefore, counted the number branches emerging from the leading edge of hyphal colonies of wild-type and Δ*tea4* mutants and discovered that branching was significantly reduced in the genetic absence of Tea4 (Figure 4I). Furthermore, Δ*tea4* mutants contained significantly fewer septa throughout their hyphae (Figure 4J). Interestingly, overexpression of the Tea4 orthologue, TeaC, in *A. nidulans* repressed septation (Higashitsuji *et al.*, 2009), while Δ*tea1* mutants in *M. oryzae* branched more frequently than the wild-type. Thus, Tea4, and the Tea4-dependent localization and retention of Tea1 at the cortex of hyphal septa are important for initiating lateral branching in *M. oryzae*.

Taken together, our data allow an improved model for cytoskeleton-mediated appressorium morphogenesis and remodeling by one of the world’s most destructive crop pathogenic fungi. First, the transition from polarized to isotropic expansion involves the redistribution of Tea1, and likely other polarity determinants, from the germ tube’s apical membrane to discrete nodes at the periphery of the septin disk. Upon reaching an appressorium turgor threshold, actin is nucleated and polymerized from Tea1-containing polarity nodes in a Tea4-depdendent manner, likely via the activation of formins. F-actin is then organized into a contractile ring, which mediates septin disk-to-ring remodeling, necessary for proper appressorium repolarization and functionality (Supplemental Figure S1A). In hyphae, Tea1 is recruited to the cortex of forming septa in a Tea4-depdendent manner, where, analogous to its role in the appressorium, we speculate that it may promote formation of the cytokinetic contractile ring driving plasma membrane ingression. Tea1’s retention at completed septa then serves as a localized pool with which to seed new polarity axis during branching (Supplemental Figure S1B). Importantly, improved understanding of the cellular control of hyphal branching may lead to new strategies to control filamentous fungal pathogens.

## MATERIALS AND METHODS

Request a protocol through *Bio-protocol*.

### Fungal culture

*M. oryzae* strains were cultured and stored as previously described (Molinari and Talbot, 2022). *M. oryzae* plate cultures were maintained on complete medium (CM) at 25°C with a 12-h light:12-h dark cycle for 7–12 d for conidium-based microscopy and 2–5 d for hyphae-based microscopy. Desiccated filter stocks were stored at –20°C and used to regenerate strains for no more than two rounds of subculture. For imaging experiments, conidia were isolated from seven to 10-day-old plate cultures in sterile water, passed through Miracloth (EMD Millipore), and washed twice by centrifugation to remove debris and traces of medium. Conidia were counted using a hemocytometer and resuspended in sterile water to a concentration of 5 × 10^4^ conidia/ml in 350 μl of sterile water. Conidial suspensions were pipetted into eight-well Nunc Lab-Tek chambers (Thermo Fisher Scientific) and allowed to adhere to the borosilicate cover glass undisturbed for ∼ 30 min before the start of imaging. Hyphae from the growing edge of a plate culture were excised from CM plates and placed inverted on the cover glass of a FluoroDish (World Precision Instruments) for imaging.

### Strain construction

High-fidelity Phusion polymerase (Apex Bio) was used to amplify linear Polymerase chain reaction (PCR) products from genomic DNA, and oligonucleotides were designed using SnapGene software (version 6.2.1, GSL Biotech). Genomic DNA sequences were retrieved from the *M. oryzae* database (http://fungi.ensembl.org/Magnaporthe_oryzae/Info/Index). DNA constructs for targeted gene replacements and protein tagging was assembled using either In-Fusion cloning (Clontech Laboratories) or yeast gap-repair. To generate *TEA4* (MGG_06439) and *SEP3* (MGG_01521) gene replacement constructs, two ∼1 kb fragments flanking the respective open reading frames were amplified from genomic DNA isolated from a wild type strain Guy11, and assembled as flanks of a hygromycin resistance cassette (HYG), amplified from plasmid pCB1004 (Sweigard *et al.*, 1997), in a uracil auxotrophic yeast gap-repair plasmid.

### Fungal Transformation

Polythene glycol-mediated genetic transformation of *M. oryzae* protoplasts was carried out as previously described (Talbot *et al.*, 1993). To generate strains expressing fluorescent fusion proteins, protoplasts were transformed with plasmids designed to integrate into the genome at ectopic sites and expression was confirmed via fluorescence microscopy. To generate the Δ*tea4* and Δ*sep3* mutants, a split marker strategy was employed (Catlett *et al.*, 2003). Protoplasts of entry strains were cotransformed with two PCR products derived from full-length gene replacement constructs, consisting of 1 kb sequences upstream or downstream of the target site, fused to complementary halves of a HYG sequence. Confirmation of site-specific homologous integration of the gene deletion and fusion constructs was obtained through PCR using primers positioned upstream and downstream of the expected integration sites, but outside of the constructs.

### Microscopy and Image Analysis

Fluorescence microscopy was performed on a Nikon Ti-E Eclipse inverted microscope outfitted with a 100 × 1.49 N.A oil immersion Apo TIRF Nikon objective, a Perfect Focus System (Nikon), and a motorized Piezo stage (Nikon). Calcofluor white, eGFP, and RFP-T were detected using Zyla 4.2 sCMOS camera (Andor Technology). Fluorophores were excited at 405 nm, 485 nm, and 560 nm using an AURA II solid-state triggered illuminator. Z-series were acquired at 0.25-µm intervals. Images were viewed and hardware was controlled using NIS-Elements AR software (version 4.60). Cells were imaged at room temperature (21–23°C). Where applicable, data sets were deconvolved with spherical aberration correction and background subtraction under “automatic” setting for three-dimensional or two-dimensional datasets using NIS Elements AR software. Imaris 9.5.1 (Bitplane) was used to generate maximum-intensity projections and the “Snapshot” function was used to generate tag image file format files (TIFs). Image manipulations were performed in IMARIS 9.5.1 (Bitplane), ImageJ software (version 2.0; National Institute of Health), and Photoshop CC software (version 22.1 Adobe).

### Line-scan analysis

Line scans through fungal cells were performed in Fiji using the “fixed length line tool” macro and the “plot profile” functions. For line scans through the apex of hyphae and germ tubes, the center of the fixed line was placed at the cell tip, and the length was set to 5 µm. For appressoria, the center of the line was placed in the middle of the appressorium and extended for 10 µm. Fluorescence intensity over time was extracted from time series using the “plot Z-axis profile” tool. Background subtraction was applied to Tea1-GFP fluorescence intensity values in wild-type and Δ*tea4* septation time lapse sequences to control for the increased cytoplasmic fluorescence in Δ*tea4* hyphae. Briefly, fluorescence intensity values from 2 × 2 pixel regions, draw adjacent to each septation event, were subtracted from the Tea1-GFP fluorescence intensity values measured at each analyzed septum. Line scans were performed on nondeconvolved max intensity projections of fluorescence micrographs. Fluorescence intensity values were exported to Excel (Microsoft, version 16.16.27) and plotted in Prism 9 (GraphPad, version 9.5.1).

### DNA isolation and extraction

Genomic DNA was extracted from cultured mycelia using the Cetyl Trimethyl Ammonium Bromide method as previously described (Molinari and Talbot, 2022). PCR was carried out using Taq polymerase (Genscript) followed by standard gel electrophoresis. Restriction enzyme digests were performed using Anza restriction enzymes following the associated protocol (Thermo Fisher Scientific). PCR amplicons were purified using Wizard SV Gel and PCR clean-up System, and plasmids were isolated from *Escherichia coli* liquid culture using the Wizard Plus SV Miniprep DNA purification system (Promega).

### Detached leaf pathogenicity assays and lesion quantification

Three rice (*Oryza sativa*) seedlings of cultivar Yt16 (Farman, University of Kentucky) were grown from seed in ProMix LP15 Multipurpose potting mix (Premier Horticulture) in 4 × 4-inch pots for 3 wk with Chelate Iron supplementation (Sprint 330). Three detached leaves from different plants were placed in petri dishes containing 0.8% water agar. Conidia were harvested from 10-day-old cultures of *M. oryzae* with sterile water, washed twice, and resuspended in 0.2% gelatin solution. Conidia were counted using a hemocytometer, and concentrations were normalized to 1 × 10^5^ conidia/ml. Five 20 ul droplets were inoculated onto each detached leaf and incubated at room temperature (21–23°C) for 7 d. Leaves were then taped onto white paper and scanned using an Epson Perfection V850 Pro (Digital ICE technologies) at 720 pixels per inch.

Images were uploaded to FIJI (ImageJ) and the “Set Scale” function was used to convert pixels to centimeters. Lesions were cropped from images and the “Split Channels” function was applied. The Red channel was selected for further analysis due to the highest degree of contrast between healthy and diseased tissue. A Gaussian Blur Filter was applied to the image with a sigma radius of 1.0. The “set color threshold” function was used to define the lesion in the color image. Binary threshold was set using the “adjust threshold” tool. Small pixel holes were filled using the “fill holes” function under the “binary” process. Lesion was measured using the “analyze particles” function with additional “outlines” image output to verify the area measured. Each lesion was measured, and the average lesion size was plotted and analyzed in Prism 9 (Graphpad, version 9.5.1). A *t* test was performed between the wild-type and mutant treatments. Two replicates were performed, with six leaves analyzed per replicate.

### Hyphal straightness and speed

Fluorescence images were acquired every 3 min for 3 h and datasets uploaded to IMARIS 9.5.1 (Bitplane). The “spot detection” tool was used to track Tea1-GFP-labeled hyphal tips over time. The GFP channel was selected for analysis with an estimated spot diameter of 2.89 μm with “background subtraction”. Spot quality was set at above 45.9 and tracking conducted with the “autoregressive motion” algorithm. Track duration was filtered to above 300 s. Any nontip specific detected spots were manually erased. The tracks were analyzed under the “statistics” tab and “selection” filters. The statistical data requested was “track straightness” and “track mean speed.”

### Branching and septation analysis

Hyphae growing on agar pads were stained with 3 ng/ml calcofluor white for 10 min in Fluorodishes (World Precision Instruments) then washed with water before being imaged by fluorescence microscopy. Maximum intensity projections of individual hyphae were measured in Fiji using the “segmented line” and “measure” tool, and the number of branches and septa occurring along their length were counted manually. Data was plotted in Prism 9 (Graphpad, version 9.5.1) and a nonlinear regression was performed with the fit forced through *x* = 0, *y* = 0.

### Statistical analyses

No statistical methods were used to predetermine sample size. All statistical significance testing was performed in Prism 9 (GraphPad, version 9.5.1)

## Supplementary Material





## Abbreviations used:

CM: complete medium
DNA: deoxyribonucleic acid
F-actin: filamentous actin
GFP: green fluorescent protein
PCR: polymerase chain reaction
RFP: red fluorescent protein
sCMOS: scientific complementary metal-oxide-semiconductor
SH3: Src homology 3
Tea: tip elongation aberrant
TIF: tagged image file format
