# Supplementary material for: The cell-end protein Tea4 spatially regulates hyphal branch initiation and appressorium remodeling in the blast fungus Magnaporthe oryzae
Source: Mol Biol Cell. 2023 Dec 14;35(1):br2. doi: 10.1091/mbc.E23-06-0214 (PMC10881174; doi:10.1091/mbc.E23-06-0214)
Supplement: Supplementary file 4 [file mbc-35-br2-s001.pdf]

Supplementary Materials

Molecular Biology of the Cell

Rogers *et al*.

Figure 5. Rogers, Taylor, and Egan

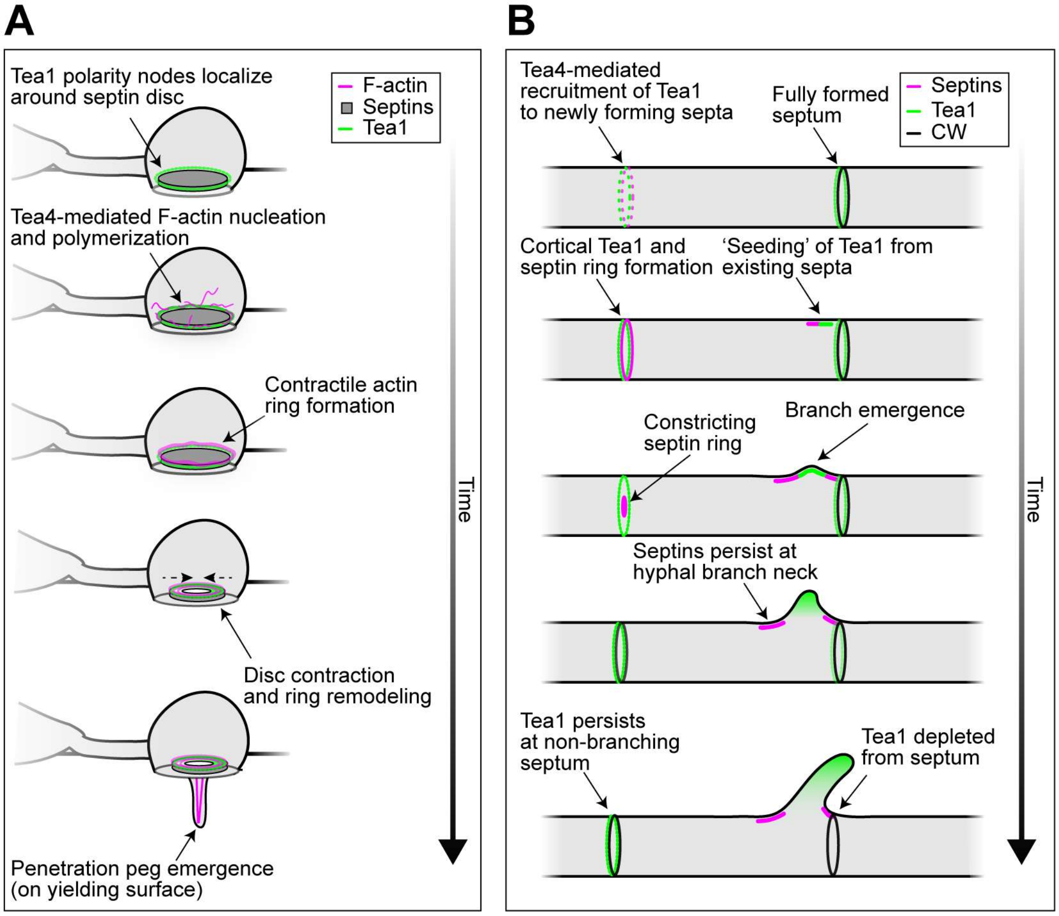

Figure S1. Tentative model for the roles of Tea1-Tea4 during appressorium morphogenesis and hyphal branching by the blast fungus *M. oryzae*. (A) During appressorium expansion, Tea1-containing polarity nodes localize around the periphery of the cortical septin disc. Tea4, which is likely also present within these nodes, promotes the nucleation and polymerization of F-actin, which assembles into a contractile ring. Constriction of the F-actin ring promotes septin disc-to-ring remodeling and

appressorium repolarization, essential for the emergence of a penetration hyphae (peg) from its base. (B) In polarized hyphae, Tea1 localizes to a ring at the cortex of forming septa in a Tea4-mediated manner, and is likely scaffolded by septins. Here, Tea1 promotes formation of the actomyosin cytokinetic ring to drive plasma membrane ingression. Unlike the septin ring, the Tea1 ring does not constrict and instead remains cortical at non-branching septa. Following branch ‘activation’, likely by upstream signaling components, cortically localized Tea1 redistributes from the septum to a site immediately below it, where septins also assemble, possibly to induce membrane curvature. Upon subsequent branch emergence, Tea1 localizes to the polarized branch hyphal tip, while septins persist at the branch neck.
