# Supplementary material for: The cell-end protein Tea4 spatially regulates hyphal branch initiation and appressorium remodeling in the blast fungus Magnaporthe oryzae
Source: Mol Biol Cell. 2023 Dec 14;35(1):br2. doi: 10.1091/mbc.E23-06-0214 (PMC10881174; doi:10.1091/mbc.E23-06-0214)
Supplement: Supplementary file 5 [file mbc-35-br2-s002.pdf]

The cell-end protein Tea4 spatially regulates hyphal branch initiation and appressorium remodeling in the blast fungus Magnaporthe oryzae

Martin Egan, Audra Rogers, and Rachel Taylor

Corresponding author(s): Martin Egan, University of Arkansas Systems Divison of Agriculture

Review Timeline:

Submission Date:	2023-06-06
Editorial Decision:	2023-07-07
Revision Received:	2023-08-16
Editorial Decision:	2023-09-19
Revision Received:	2023-09-28
Accepted:	2023-10-16

Editor-in-Chief: Matthew Welch

Transaction Report:

(Note: With the exception of the correction of typographical or spelling errors that could be a source of ambiguity, letters and reports are not edited. The original formatting of letters and referee reports may not be reflected in this compilation.)

RE: Manuscript #E23-06-0214
TITLE: The cell-end protein Tea4 spatially regulates hyphal branch initiation and appressorium remodeling in the blast fungus Magnaporthe oryzae

Dear Dr. Egan:

The reviewers appreciate that the quality of data and results of your study will be useful to understanding fungal morphogenesis. However, both reviewers raised major concerns about presentation, writing, and experimental analysis. A revision can be considered and will be re-reviewed but needs substantial revision to be suitable for publication.

Sincerely,

Amy Gladfelter
Monitoring Editor
Molecular Biology of the Cell

Dear Dr. Egan,

The review of your manuscript, referenced above, is now complete. The Monitoring Editor has decided that your manuscript is not acceptable for publication at this time, but may be deemed acceptable after specific revisions are made, as described in the Monitoring Editor's decision letter above and the reviewer comments below.

A reminder: Please do not contact the Monitoring Editor directly regarding your manuscript. If you have any questions regarding the review process or the decision, please contact the MBoC Editorial Office (mboc@ascb.org).

When submitting your revision include a rebuttal letter that details, point-by-point, how the Monitoring Editor's and reviewers' comments have been addressed. (The file type for this letter must be "rebuttal letter"; do not include your response to the Monitoring Editor and reviewers in a "cover letter.") Please bear in mind that your rebuttal letter will be published with your paper if it is accepted, unless you have opted out of publishing the review history.

Authors are allowed 90 days to submit a revision. If this time period is inadequate, please contact us at mboc@ascb.org.

Revised manuscripts are assigned to the original Monitoring Editor whenever possible. However, special circumstances may preclude this. Also, revised manuscripts are often sent out for re-review, usually to the original reviewers when possible. The Monitoring Editor may solicit additional reviews if it is deemed necessary to render a completely informed decision.

In preparing your revised manuscript, please follow the instruction in the Information for Authors (www.molbiolcell.org/info-for-authors). In particular, to prepare for the possible acceptance of your revised manuscript, submit final, publication-quality figures with your revision as described.

To submit the rebuttal letter, revised manuscript, and figures, use this link: Link Not Available

MBoC offers the option to publish your work with immediate open access. Open access can increase the discoverability and usability of your research. If you would like to publish your paper with immediate open access but did not select this option during initial submission, please contact the editorial office at mbc@ascb.org.

Thank you for submitting your manuscript to MBoC. We look forward to receiving your revised paper.

Sincerely,

Eric Baker
Journal Production Manager
MBoC Editorial Office
mbc@ascb.org

Reviewer #1 (Remarks to the Author):

Rogers et al investigated septa, branches and appressoria in Magnaporthe oryzae focusing on cell end marker proteins, septins and actin. The authors used fluorescently labeled Tea1, Sep5 and actin (Life Act) in WT and strains deleted for Tea 4 or Sep3. The microscopy and associated quantitative analyses are excellent. The very clear finding that Tea1 associates with septins and persists at nonbranch associated septa, but rapidly relocalizes in branch-associated septa gives important clues to the hyperbranching phenotype seen for septin deletion strains in many fungi. Evidence for the role of Tea4 in both appressorium formation and branching is also noteworthy.

I felt it was too difficult to pull the important findings out of the paper. This might be a side effect of the very condensed format, but just a little more context and interpretation would help the reader a lot. For example, because the authors jump between multiple developmental stages it would be helpful to have a diagram showing appressorium formation from conida versus septation and branching in vegetative hyphae. It would also be useful for the authors to explicitly state how they think Tea4, Tea1, septins, and actin are interacting. While I appreciate the authors' caution on speculating beyond the data, a model or maybe just a timeline of which proteins show up when and where would really help make these strong findings more clear, especially to those who might not be as familiar with the M. oryzae system.

A few typos noted:
1. "While Sep5-RFP remained at sites of membrane curvature at the branch neck, Tea1-GFP localized to the new hyphal apex (Fig H-J)." And following sentence...Figure number missing
2. "We therefore deleted the gene encoding for the core septin, Sep3, in a strain co-expressing Sep5-RFP and Tea1-GFP an imaged appressorium development in this mutant relative to wildtype." Correct to: "Tea1-GFP and imaged..."
3. "Interestingly, Lifeact-RFP-labelled F-actin failed accumulate within..." Correct to: "to accumulate"
4. Fig 3. J panel in fig is missing in legend. K not in Fig or legend.
5. Fig 4 A Legend doesn't specify fluorescence monitored (Tea1? from context).

Reviewer #2 (Remarks to the Author):

In this work, Rogers et al. describe the temporal localization of Tea1, Septins and actin filaments in the blast fungus M. oryzae. This fungus assembles different structures including hypha and infection cells called appressoria. The study involves genetically modified strains that express fluorescently labeled proteins of interest (expressed from constructs integrated at ectopic genomic sites) in both wild-type controls and in Δtea4 or Δsep3 genetic backgrounds.
The manuscript lacks the discussions of the results, which would be expected to be interwoven with the results in the selected manuscript format. The authors draw few conclusions from their results and do not interpret the potential meanings of their findings. Therefore, without discussing the results within the greater context of the field, the significance of the work is unknown. In addition, the introduction does not contain sufficient information about the field and about this fungus. Finally, the figures need better labeling.
I offer some suggestions below to improve this manuscript and prepare it for a general cell biology readership.

Major issues:

1- This manuscript requires the authors to write down the conclusions for all the results and interpret the meaning of their findings. These conclusions/interpretations should either be interwoven with the results for a "results and discussion" section OR written in a distinct "discussion" section.

2- The data appears to be of good quality, however for many graphs, the N value is not reported on the graph or in the legends/methods. For this reason, this reviewer found that the manuscript does not meet the MBoC "data presentation" requirements.

3- More information about the field is required in the introduction section. The authors need to explain what was previously known about Tea1, Tea4, actin and septins in fungi and why the experiments they proposed were needed in the field.

4- Some figure panels appear to be redundant and don't seem to add to the story.
For example, Figure 2B doesn't add any information. Figure 2A is sufficient for the information written in the text.

5- The figure panels are called out of order in the text.

6- Figure panels lack labels to emphasize the desired feature(s). For example:
- Where is the Septin ring in Figure 1A?
- Where is the branch neck in Figures 2H, I and J?
- What is different between wildtype and Δsep3 in Figure 3A?
- What's unusual about the cell in Figure 3C (show a wildtype as a comparison)?
- What's different between wildtype and Δtea4 in Figure 3I? It looks like the wildtype is a cross-section and the mutant is not.

- Should we pay attention to the brown spots in Figure 3N? Are those leaves (not mentioned in the legend)?
- What features should we look at in Figure 4D? These images are of poor quality (too blurry for publication) compared to the other images in the manuscript.
- "Interestingly, while Tea1-GFP retained a nodal ring-like confirmation in the absence of a septin disc, Tea1-GFP also mislocalized to aberrant patches on the appressorium cortex (Fig.3A)."
Label the "aberrant patches" in the image.

7- The information shown in the figure panel does not always represent the information written in the text where the figures is called. For example:
"However, unlike Sep5-RFP, which rapidly delocalized from septation sites, presumably following the completion of cytokinesis, Tea1-GFP persisted indefinitely at newly formed septa (Fig.2.C-H,K)."
Figure 2H does not show that Tea1-GFP persists at the septum. Tea1-GFP in that cell leaves the septum.
8- The trace plots lack examples of the position where the fluorescence intensity was measured. Show one example line trace and state whether other measurements were taken at the same position.

9- The introduction includes a detailed explanation of the morphology of this fungus. An accompanying diagram, even in the supplemental materials, would really help understand the different structures of this interesting fungus.

Minor points:
1- What does "hpi" stand for (legend of Figure 1)?
2- "We set out to gain new insight into the behavior of polarity determents during the transition from polarized to isotropic growth that occurs during the differentiation of appressoria."
Do you mean determinants?
3- "Interestingly, components of the exocyst complex, which mediates the tethering of post- Golgi secretory vesicles to the plasma membrane for polarized exocytosis, localize in a similar ring confirmation (Gupta et al., 2015)."

"Interestingly, while Tea1-GFP retained a nodal ring-like confirmation in the absence of a septin disc, Tea1-GFP also mislocalized to aberrant patches on the appressorium cortex (Fig.3A)."
Do you mean conformation?
4- "Consistent with its known role as a cell-marker (Dagdas et al., 2012; Qu et al., 2022), Tea1-GFP localized to the leading edge of apically expanding germ tubes and was enriched at the equatorial plane (Fig.1A)."
Do you mean cell-end marker?
5- "Consistent with previous studies of both tea4 (Patkar et al., 2010), and tea1 mutants in M. oryzae (Qu et al., 2022), tea4 conidia produced extremely long germ tubes (Fig.3C,E), whose apices contained a small but significant increase in Tea1-GFP compared to wildtype (Fig.3D)."
What are conidia? Use that term in the introduction.
6- While Sep5-RFP remained at sites of membrane curvature at the branch neck, Tea1-GFP localized to the new hyphal apex (Fig H-J).
Figure number is missing.

Dear Dr. Gladfelter,

Many thanks for overseeing the review of our manuscript "The cell-end protein Tea4 spatially regulates hyphal branch initiation and appressorium remodeling in the blast fungus *Magnaporthe oryzae"*.

We have now submitted a revised version of this manuscript, addressing each of the reviewer's concerns. We sincerely thank the reviewers for their constructive critiques, which have greatly improved the manuscript.

Please see our point-by-point responses below, in green text.

Reviewer #1 (Remarks to the Author):

Rogers et al investigated septa, branches and appressoria in Magnaporthe oryzae focusing on cell end marker proteins, septins and actin. The authors used fluorescently labeled Tea1, Sep5 and actin (Life Act) in WT and strains deleted for Tea 4 or Sep3. The microscopy and associated quantitative analyses are excellent. The very clear finding that Tea1 associates with septins and persists at nonbranch associated septa, but rapidly relocalizes in branch-associated septa gives important clues to the hyperbranching phenotype seen for septin deletion strains in many fungi. Evidence for the role of Tea4 in both appressorium formation and branching is also noteworthy.

I felt it was too difficult to pull the important findings out of the paper. This might be a side effect of the very condensed format, but just a little more context and interpretation would help the reader a lot.
Yes, we struggled a little to include everything we wanted to say within the 'Brief Reports' format. We hope the new material helps with this aspect.
For example, because the authors jump between multiple developmental stages it would be helpful to have a diagram showing appressorium formation from conida versus septation and branching in vegetative hyphae.
We agree with the reviewer that it was probably a bad idea to jump between developmental stages and have revised the order of the figures, and included new cartoons, to make things easier to follow.
It would also be useful for the authors to explicitly state how they think Tea4, Tea1, septins, and actin are interacting. While I appreciate the authors' caution on speculating beyond the data, a model or maybe just a timeline of which proteins show up when and where would really help make these strong findings more clear, especially to those who might not be as familiar with the M. oryzae system.
Many thanks for the suggestion. We have generated a new 'model' figure (Fig.5) to highlight and summarize what we think is going on.

A few typos noted:
1. "While Sep5-RFP remained at sites of membrane curvature at the branch neck, Tea1-GFP localized to the new hyphal apex (Fig H-J)." And following sentence...Figure number missing

Fixed, thank you.
2. "We therefore deleted the gene encoding for the core septin, Sep3, in a strain co-expressing Sep5-RFP and Tea1-GFP an imaged appressorium development in this mutant relative to wildtype." Correct to: "Tea1-GFP and imaged..."
Thanks for spotting this type – now corrected in the text.
3. "Interestingly, Lifeact-RFP-labelled F-actin failed accumulate within..." Correct to: "to accumulate"
Thanks, we have now corrected this.
4. Fig 3. J panel in fig is missing in legend. K not in Fig or legend.
Fixed, thank you.
5. Fig 4 A Legend doesn't specify fluorescence monitored (Tea1? from context).
Thank you, this has been revised in the figure.

Reviewer #2 (Remarks to the Author):

In this work, Rogers et al. describe the temporal localization of Tea1, Septins and actin filaments in the blast fungus M. oryzae. This fungus assembles different structures including hypha and infection cells called appressoria. The study involves genetically modified strains that express fluorescently labeled proteins of interest (expressed from constructs integrated at ectopic genomic sites) in both wild-type controls and in ∆tea4 or ∆sep3 genetic backgrounds.
The manuscript lacks the discussions of the results, which would be expected to be interwoven with the results in the selected manuscript format.The authors draw few conclusions from their results and do not interpret the potential meanings of their findings. Therefore, without discussing the results within the greater context of the field, the significance of the work is unknown.
Many thanks for the constructive comments on our manuscript. We agree that our original submission was lacking some clearly articulated conclusions and have now added these to the Results and Discussion section as well as creating a new 'model' figure to interpret the potential meanings of our data.
In addition, the introduction does not contain sufficient information about the field and about this fungus.
To address this critique, we have made a new panel within Figure 1., providing additional information about appressorium morphogenesis.
Finally, the figures need better labeling. Thank you for the suggestion. We have added further labeling to figures.
I offer some suggestions below to improve this manuscript and prepare it for a general cell biology readership.

Major issues:

1- This manuscript requires the authors to write down the conclusions for all the results and interpret the meaning of their findings. These conclusions/interpretations should either be interwoven with the results for a "results and discussion" section OR written in a distinct "discussion" section.

Many thanks for this important suggestion. We were struggling with the tight character count of this ‘Brief Report’ format and excluded important concluding statements for each section. We have now revised the text in the manuscript.

2- The data appears to be of good quality, however for many graphs, the N value is not reported on the graph or in the legends/methods. For this reason, this reviewer found that the manuscript does not meet the MBoC "data presentation" requirements.
Many thanks for pointing this out. We agree that this is a vital concern and have reported N values on each plot.

3- More information about the field is required in the introduction section. The authors need to explain what was previously known about Tea1, Tea4, actin and septins in fungi and why the experiments they proposed were needed in the field.
We appreciate this suggestion and agree with the reviewer. However, we have very limited space in this ‘Brief Report’ format to provide exhaustive background and feel that, with our revised content, there is sufficient background to understand the study and its significance.

4- Some figure panels appear to be redundant and don't seem to add to the story. For example, Figure 2B doesn't add any information. Figure 2A is sufficient for the information written in the text.
We have reorganized this figure.

5- The figure panels are called out of order in the text.
Thank you – we have now fixed this.

6- Figure panels lack labels to emphasize the desired feature(s). For example:
- Where is the Septin ring in Figure 1A?
We have added cartoons in Fig.1A that should make this much more apparent.
- Where is the branch neck in Figures 2H, I and J?
We have added an arrow and label to Fig.3G to highlight what we mean by ‘branch neck’.

- What is different between wildtype and Δsep3 in Figure 3A?
The Δsep3 mutant is unable to form higher-order septin structures, and therefore in this mutant, Sep5-RFP does not localize to a disc or ring, unlike wildtype. We wanted to understand what impact this had on Tea1-GFP localization. We quantify this phenotype in the subsequent panel.

- What's unusual about the cell in Figure 3C (show a wildtype as a comparison)?
We have removed this particular micrograph from the figure. It was showing a mutant conidium with two germ tubes, rather than the typical one. We have retained text in the manuscript that describes and quantifies this.
- What's different between wildtype and Δtea4 in Figure 3I? It looks like the wildtype is a cross-section and the mutant is not.

The differences here are a reflection of the Δtea4 phenotype alone. Both images represent maximum intensity projections of Z-series through the entire appressoria. This particular cell was selected to highlight the disorganized actin cytoskeleton. We quantified this phenotype further in an unbiassed way by preforming line scans through 22 appressoria of each strain.

- Should we pay attention to the brown spots in Figure 3N? Are those leaves (not mentioned in the legend)?

Yes, these are rice leaves with necrotic blast disease lesions. We have added this information to the legend.

- What features should we look at in Figure 4D? These images are of poor quality (too blurry for publication) compared to the other images in the manuscript.

We have added annotations to Figure 4D to aid in its interpretation, and hope that the reviewer sees some value in this panel. These images are unique within the manuscript in that they represent end-on images of hyphae, as if the viewer is looking straight into a tube, as this was deemed to be the best way to illustrate the differences between the wildtype and mutant. The proteins of interest therefore appear as a ring/circle (or not). The images represent projections of three different proteins/fluorophores imaged in multiple Z every 3 minutes for ~ 30 minutes, which is technically quite challenging considering photobleaching, XY drift, and phototoxicity issues. We apologize for the inherently lower resolution of the images (the rings are ~ 3 um in diameter), but don't necessarily agree that they are too blurry for publication. We think the features/processes are sufficiently clear in the wildtype, but are less so in the mutant, but this is due to the phenotype and the biology itself.

- "Interestingly, while Tea1-GFP retained a nodal ring-like confirmation in the absence of a septin disc, Tea1-GFP also mislocalized to aberrant patches on the appressorium cortex (Fig.3A)."

Label the "aberrant patches" in the image.

Thank you - e have done as suggested.

7- The information shown in the figure panel does not always represent the information written in the text where the figures is called. For example:

"However, unlike Sep5-RFP, which rapidly delocalized from septation sites, presumably following the completion of cytokinesis, Tea1-GFP persisted indefinitely at newly formed septa (Fig.2.C-H,K)."

Figure 2H does not show that Tea1-GFP persists at the septum. Tea1-GFP in that cell leaves the septum.

Apologies for these errors – we have corrected them is the manuscript.

8- The trace plots lack examples of the position where the fluorescence intensity was measured. Show one example line trace and state whether other measurements were taken at the same position.

Many thanks for the suggestion. We have included cartoons in Fig.1C and D, and Fig. 3C to better explain how we generated the fluorescence intensity plots.

9- The introduction includes a detailed explanation of the morphology of this fungus. An accompanying diagram, even in the supplemental materials, would really help understand the different structures of this interesting fungus.
Many thanks for the suggestion. We have included a cartoon diagram in Fig.1A to help with the interpretation of our work.

Minor points:
1- What does "hpi" stand for (legend of Figure 1)?
Hpi stands for ‘hours post inoculation’, which we’ve added to the legend.
2- "We set out to gain new insight into the behavior of polarity determents during the transition from polarized to isotropic growth that occurs during the differentiation of appressoria."
Do you mean determinants?
Yes, we did. Corrected in the text, thanks

3- "Interestingly, components of the exocyst complex, which mediates the tethering of post- Golgi secretory vesicles to the plasma membrane for polarized exocytosis, localize in a similar ring confirmation (Gupta et al., 2015)."

"Interestingly, while Tea1-GFP retained a nodal ring-like confirmation in the absence of a septin disc, Tea1-GFP also mislocalized to aberrant patches on the appressorium cortex (Fig.3A)."
Do you mean conformation?
Yes, we did. Corrected in the text, thanks.
4- "Consistent with its known role as a cell-marker (Dagdas et al., 2012; Qu et al., 2022), Tea1-GFP localized to the leading edge of apically expanding germ tubes and was enriched at the equatorial plane (Fig.1A)."
Do you mean cell-end marker?
Yes, we did. Corrected in the text, thanks.
5- "Consistent with previous studies of both tea4 (Patkar et al., 2010), and tea1 mutants in M. oryzae (Qu et al., 2022), tea4 conidia produced extremely long germ tubes (Fig.3C,E), whose apices contained a small but significant increase in Tea1-GFP compared to wildtype (Fig.3D)."
What are conidia? Use that term in the introduction.
We have included a new cartoon in Fig.1A, to introduce the *Magnaporthe oryzae* model system.
6- While Sep5-RFP remained at sites of membrane curvature at the branch neck, Tea1-GFP localized to the new hyphal apex (Fig H-J).
Figure number is missing.
Thank you for spotting this – we have corrected the typo.

RE: Manuscript #E23-06-0214R
TITLE: "The cell-end protein Tea4 spatially regulates hyphal branch initiation and appressorium remodeling in the blast fungus Magnaporthe oryzae"

Dear Martin:

Thank you for sending this revision back to MBoC. While much improved, there are still some valid concerns raised by reviewer 2 that require changes to the text and figures, though no new experiments are critical. Please ensure that you include some synthesis and interpretations as to the impact of this work on either Magnaporthe or fungal polarity more broadly. Additionally, please make sure to change all the figure panels that are highlighted by the reviewer that are not supported by the text or revise the way the results are discussed so that they are represented in the figures. I look forward to seeing these modifications!

Sincerely,
Amy Gladfelter
Monitoring Editor
Molecular Biology of the Cell

Dear Dr. Egan,

The review of your manuscript, referenced above, is now complete. The Monitoring Editor has decided that your manuscript requires minor revisions before it can be published in Molecular Biology of the Cell, as described in the Monitoring Editor's decision letter above and the reviewer comments (if any) below.

A reminder: Please do not contact the Monitoring Editor directly regarding your manuscript. If you have any questions regarding the review process or the decision, please contact the MBoC Editorial Office (mboc@ascb.org).

When submitting your revision include a rebuttal letter that details, point-by-point, how the Monitoring Editor's and reviewers' comments have been addressed. (The file type for this letter must be "rebuttal letter"; do not include your response to the Monitoring Editor and reviewers in a "cover letter.") Please bear in mind that your rebuttal letter will be published with your paper if it is accepted, unless you have opted out of publishing the review history.

If your manuscript contains a Significance Statement, please make sure that it consists of three separate bullet points totaling a maximum of 100 words and addressing each of the following points (see https://www.molbiolcell.org/curation-tools):
First bullet: What is the background context? What gap in knowledge does this study addres?
Second bullet: What are the key findings? What is unique or new about the approach?
Third bullet: Why is this paper significant? How might it influence future research?

Authors are allowed 15 days to submit a revision. If this time period is inadequate, please contact us immediately at mboc@ascb.org.

In preparing your revised manuscript, please follow the instruction in the Information for Authors (www.molbiolcell.org/info-for-authors). In particular, to prepare for the possible acceptance of your revised manuscript, submit final, publication-quality figures with your revision as described.

To submit the rebuttal letter, revised version, and figures, please use this link (please enable cookies, or cut and paste URL): Link Not Available

MBoC offers the option to publish your work with immediate open access. Open access can increase the discoverability and usability of your research. If you would like to publish your paper with immediate open access but did not select this option during initial submission, please contact us at mbc@ascb.org..

Authors of Articles and Brief Communications whose manuscripts have returned for minor revision ("revise only") are encouraged to create a short video abstract to accompany their article when it is published. These video abstracts, known as Science Sketches, are up to 2 minutes long and will be published on YouTube and then embedded in the article abstract. Science Sketch Editors on the MBoC Editorial Board will provide guidance as you prepare your video. Information about how to prepare and submit a video abstract is available at www.molbiolcell.org/science-sketches. Please contact mboc@ascb.org if you are interested in creating a Science Sketch.

Thank you for submitting your manuscript to MBoC. Please do not hesitate to contact this office if you have any questions.

Sincerely,

Eric Baker
Journal Production Manager
MBoC Editorial Office
mbc@ascb.org

Reviewer #1 (Remarks to the Author):

In this revised manuscript Rogers et al investigated septa, branches and appressoria in Magnaporthe oryzae focusing on cell end marker proteins, septins and actin via fluorescently labeled Tea1, Sep5 and actin (Life Act) in WT and strains deleted for Tea 4 or Sep3. The revision does a great job of putting the excellent microscopy findings into context. My only criticism is that the model shown in Figure 5 needs to be explained. It feels like the last paragraph of the paper bringing together the pieces is missing. Similarly, the legend for Figure 5 should explain the model. If word limit is a concern, perhaps the authors could add a couple of sentences to the body of the paper and then go into more detail in the Figure legend.

Reviewer #2 (Remarks to the Author):

In this work, Rogers et al. describe the temporal localization of Tea1, Septins and actin filaments in the blast fungus M. oryzae. This fungus assembles different structures including hypha and infection cells called appressoria. The study involves genetically modified strains that express fluorescently labeled proteins of interest (expressed from constructs integrated at ectopic genomic sites) in both wild-type controls and in Δtea4 or Δsep3 genetic backgrounds.
I want to thank the authors for their improvements to the manuscript. I appreciate the diagrams in Figures 1A and 5 as well as the improvements made to the other figure panels. Although improved, this updated version of the manuscript still lacks interpretations and some figures still do not support the claims made in the text. Below, I include suggestions to improve these remaining deficiencies.

Major concerns:

Lack of interpretation of the results and lack of integration of the findings within the field of cellular polarity establishment in fungal cells.
1- This work investigates the role of Tea1 and Tea4 in the growth of the appressorium and hyphal cell. We have quite a bit of information about the Tea1/Tea4 complex from other fungal model organisms. In these other models, the Tea1/Tea4 complex is critical for cellular polarity establishment. This complex localizes to sites of cell growth in a microtubule-dependent manner where it recruits formins for local actin polymerization. Loss of tea4 in fission yeast results in aberrant localization of Tea1 and defective cell growth. It's hard to understand the significance of this work for a few reasons.
First, the manuscript begins with a detailed description of the development of the appressorium suggesting that this will be the focus of the work. However, it is not. The polarity complex Tea1/Tea4 is the focus of this work. Restructuring the introduction so it starts with defining the topic of the research presented here would help authors understand its significance.
Second, the observations of the role of the Tea1/Tea4 complex in the appressorium and hyphal cells are not discussed. There is no interpretation of the data. To address this void, please answer the following question. What do your findings in the appressorium/hyphal cells of M. oryzae mean to the greater field of cellular polarity establishment in fungi?
The last sentence of your abstract states "Together, our data provide new insight into polarity remodeling during infection-related and vegetative growth by the blast fungus." What is this new insight and what interpretations can you derive from it? In other words what do your findings about the Tea1/Tea4 complex mean to our knowledge of the development of the appressorium/hyphal cells of M. oryzae?
2- There's a disconnect between the two halves of the manuscript. The first half investigates the role of Tea1 and Tea4 in the appressorium while the second half investigates these proteins in hyphal cells. Yet, there is no final statement connecting the two halves of this work leaving this manuscript a disjointed list of observations. What's the general role of the Tea1/Tea4 complex in M. oryzae? Does it have two different roles that will need to be determined in the future?
The last sentence "Taken together, our data allow a tentative model for the role of cell-end proteins in the remodeling of specialized plant-infective cell types and the spatial control of branching in a model phytopathogenic fungus (Fig.5)." abruptly ends the manuscript with no interpretation of the findings. Figure 5 is a nice summary of the observations, but it is not discussed in the text and its legend is a single sentence.

Figures do not represent the claims made in the text.

3- Figure 1A. Although this diagram is a great addition, it only shows the septin ring, not the septin disc and therefore lacks the septin disc to ring transition, a main event studied in this work. Can you please modify it so that it shows the septin disc transitions to a septin ring?

4- Figure 1B. All timepoints show the septin signal as a disc that fills the cortex. What time point do you consider the septin signal to be a "ring"? This misrepresentation maybe due to image processing or the selection of a non-representative example. Please find an example that shows the septin ring structure you describe in the text.

5- Figure 2A. In the selected image, the Tea1-GFP signal is comparable between the wildtype and Δsep3 samples. The only noticeable difference between the two genotypes is the size of the Tea1-GFP disc at 6 and 14 hpi. This image does not represent the claims made in the text. Either change the claims in the text or find an image that illustrates your claim. This problem may be due to the overlapping colors in the wildtype image. Separating the colors in the wildtype panel may reveal the appearance of the Tea1-GFP for proper comparison with the Δsep3.

6- Figure 2I and J show that the fluorescence intensity of Lifeact-RFP and Tea1-GFP are lower in Δtea4 samples. However, the image shown in Figure 2G shows comparable fluorescence intensity for both markers. I assume that the Δtea4 image in 2G was processed to enhance the signal. If so, please state this difference in processing in the figure legend. If not, where does this quantification come from? How can these two images be so similar while the quantification of fluorescence intensity show such a decrease in signal in the mutant?

7- Figure 3H and 3I are confusing and don't add anything to the information shown in Figure 3G. I suggest deleting these panels to avoid confusion.

8- Figure 3J does not illustrate the claim made in the text where the figure panel is mentioned. The sentence in question is "Unlike in non-branch associated septa, where cortical Tea1-GFP persists indefinitely (Fig.3B-F,J)." Figure 3J does not show indefinite Tea1-GFP maintenance. This figure panel should be deleted.

Possible mistake in a sentence:

9- Please verify the following sentence: "Thus, the redistribution of Tea1-containing polarity nodes to the periphery of cortical septin discs proceeds septin disc-to-ring remodeling, and these nodes spatiotemporally colocalize with sites of F-actin nucleation."
Did you mean "precedes" as in "it happens before"?

Errors related to the movies.

10- This manuscript file contains 4 movie files yet only 3 are referenced in the text.

11- The movies are called out of order starting with Movie S1, S3 and S2.
12- "Interestingly, Lifeact-RFP-labelled F-actin failed to accumulate within the appressoria of Δtea4 mutants to the same extent that it did in wildtype cells during their maturation (Fig.2E), and furthermore, did not localize to conspicuous contractile F-actin rings (Movie S3)". I think you mean Movie S4 as it's the one movie that illustrates these events.
13- Movies S3 and S4 are too fast to appreciate the events they illustrate. Please slow them down.

Minor suggestions:
14- "Following the maturation of wildtype appressoria, Lifeact-RFP-labelled F-actin filaments decorated Tea1-GFP- labelled cortical rings and appeared tightly interwoven with these structures".
F-actin filaments is redundant. Choose "Lifeact-RFP-labelled F-actin" or "Lifeact-RFP-labelled actin filaments".

15- You have valuable timing data from your movies. If you found a way to label one event as "time 0", you could add the timing data to your diagrams. This timing information would provide valuable information for the field.

UNIVERSITY OF ARKANSAS
DIVISION OF AGRICULTURE

Department of
Entomology and Plant Pathology

UNIVERSITY OF ARKANSAS
DALE BUMPERS COLLEGE
OF AGRICULTURAL, FOOD & LIFE SCIENCES

217 Plant Science Building, University of Arkansas, Fayetteville, Arkansas 72701-1201
479-575-2445 · Fax 479-575-7601 ·

Martin J. Egan
09/27/23

Dear Amy,

Many thanks for overseeing the peer-review of our revised manuscript #E23-06-0214R, and for your patience as we figured out how to include the necessary content within this tight *Brief Reports* format. We are hopeful that we have now addressed the reviewers' comments to a satisfactory level, and appreciate the critiques which have greatly improved the manuscript. For full transparency, in addition to the changes suggested by the reviewers, we also decided to replace the wildtype image in Fig.2G, with a more typical appressorium showing very tight co-localization/overlap of the Tea1-GFP and Lifeact-RFP signal. Also, regrettably we had to remove our former Movie S1, to make space for an expanded model Figure S1. Please find our point-by-point response below (in green text).

Reviewer #1 (Remarks to the Author):

In this revised manuscript Rogers et al investigated septa, branches and appressoria in Magnaporthe oryzae focusing on cell end marker proteins, septins and actin via fluorescently labeled Tea1, Sep5 and actin (Life Act) in WT and strains deleted for Tea 4 or Sep3. The revision does a great job of putting the excellent microscopy findings into context. My only criticism is that the model shown in Figure 5 needs to be explained. It feels like the last paragraph of the paper bringing together the pieces is missing. Similarly, the legend for Figure 5 should explain the model. If word limit is a concern, perhaps the authors could add a couple of sentences to the body of the paper and then go into more detail in the Figure legend.
Thank you for the suggestions. We have moved the model figure to the supplementary material and have expanded its legend. We have also added text to the results and discussion to summarize our findings and put them into broader context.

Reviewer #2 (Remarks to the Author):

In this work, Rogers et al. describe the temporal localization of Tea1, Septins and actin filaments in the blast fungus M. oryzae. This fungus assembles different structures including hypha and infection cells called appressoria. The study involves genetically modified strains that express fluorescently labeled proteins of interest (expressed from constructs integrated at ectopic genomic sites) in both wild-type controls and in Δtea4 or Δsep3 genetic backgrounds.
I want to thank the authors for their improvements to the manuscript. I appreciate the diagrams in Figures 1A and 5 as well as the improvements made to the other figure panels. Although improved, this updated version of the manuscript still lacks interpretations and some figures still do not support the claims made in the text. Below, I include suggestions to improve these remaining deficiencies.

Major concerns:

Lack of interpretation of the results and lack of integration of the findings within the field of cellular polarity establishment in fungal cells.
1- This work investigates the role of Tea1 and Tea4 in the growth of the appressorium and hyphal

cell. We have quite a bit of information about the Tea1/Tea4 complex from other fungal model organisms. In these other models, the Tea1/Tea4 complex is critical for cellular polarity establishment. This complex localizes to sites of cell growth in a microtubule-dependent manner where it recruits formins for local actin polymerization. Loss of tea4 in fission yeast results in aberrant localization of Tea1 and defective cell growth. It's hard to understand the significance of this work for a few reasons.

First, the manuscript begins with a detailed description of the development of the appressorium suggesting that this will be the focus of the work. However, it is not. The polarity complex Tea1/Tea4 is the focus of this work. Restructuring the introduction so it starts with defining the topic of the research presented here would help authors understand its significance.
We appreciate the reviewer's critique and suggestions, and have restructured the introduction (reverting to that of our original submission) in an attempt to address this. We have also added some text to better frame the study and highlight knowledge gaps.

Second, the observations of the role of the Tea1/Tea4 complex in the appressorium and hyphal cells are not discussed. There is no interpretation of the data. To address this void, please answer the following question. What do your findings in the appressorium/hyphal cells of M. oryzae mean to the greater field of cellular polarity establishment in fungi?

We recognize our failure to interpret our findings further, and thank the reviewer for the critique. We have added the following text:
Taken together, our data allow an improved model for cytoskeleton-mediated appressorium morphogenesis and remodeling by one of the world's most destructive crop pathogenic fungi. Firstly, the transition from polarized to isotropic expansion involves the redistribution of Tea1, and likely other polarity determinants, from the germ tube's apical membrane to discrete nodes at the periphery of the septin disc. Upon reaching an appressorium turgor threshold, actin is nucleated and polymerized from Tea1-containing polarity nodes in a Tea4-depdendent manner, likely via the activation of formins. F-actin is then organized into a contractile ring, which mediates septin disc-to-ring remodeling, necessary for proper appressorium repolarization and functionality (Fig.S1A). In hyphae, Tea1 is recruited to the cortex of forming septa in a Tea4-depdendent manner, where, analogous to its role in the appressorium, we speculate that it may promote formation of the cytokinetic contractile ring driving plasma membrane ingression. Tea1's retention at completed septa then serves as a localized pool with which to seed new polarity axis during branching (Fig.S1B). Improved understanding of the spatial control of hyphal branching may lead to novel strategies to control filamentous fungal pathogens.

The last sentence of your abstract states "Together, our data provide new insight into polarity remodeling during infection-related and vegetative growth by the blast fungus." What is this new insight and what interpretations can you derive from it? In other words what do your findings about the Tea1/Tea4 complex mean to our knowledge of the development of the appressorium/hyphal cells of M. oryzae?

We recognize the vague and abrupt ending to the manuscript, and have added text to better frame the significance of our findings.

2- There's a disconnect between the two halves of the manuscript. The first half investigates the role of Tea1 and Tea4 in the appressorium while the second half investigates these proteins in hyphal cells. Yet, there is no final statement connecting the two halves of this work leaving this manuscript a

disjointed list of observations. What's the general role of the Tea1/Tea4 complex in M. oryzae? Does it have two different roles that will need to be determined in the future?

We agree with the reviewer, and have added text to the discussion and to the legend of the model figure to address this.

The last sentence "Taken together, our data allow a tentative model for the role of cell-end proteins in the remodeling of specialized plant-infective cell types and the spatial control of branching in a model phytopathogenic fungus (Fig.5)." abruptly ends the manuscript with no interpretation of the findings. Figure 5 is a nice summary of the observations, but it is not discussed in the text and its legend is a single sentence.

We apologize for the abrupt ending to the manuscript - we were fighting a losing battle against the 20,000-character limit. We have made some changes to the structure of the manuscript to free up some space, and have expanded on the concluding text in the manuscript, and in the legend of Figure 5 (now Figure S1).

Figures do not represent the claims made in the text.

3- Figure 1A. Although this diagram is a great addition, it only shows the septin ring, not the septin disc and therefore lacks the septin disc to ring transition, a main event studied in this work. Can you please modify it so that it shows the septin disc transitions to a septin ring?
We apologize for any confusion. Figure 1A shows the septin disc, which is labeled in green, as indicated in the inset key. We have added an additional label "septin disc" to make this clearer to the reader.

4- Figure 1B. All timepoints show the septin signal as a disc that fills the cortex. What time point do you consider the septin signal to be a "ring"? This misrepresentation maybe due to image processing or the selection of a non-representative example. Please find an example that shows the septin ring structure you describe in the text.

The term "ring" was first used to describe this appressorium-specific higher-order septin structure by Nick Talbot's group in their original Science manuscript, (Dagdas et al., 2012). When imaged in a single focal plane, the ring-like geometry of this structure is very obvious. However, when Z-series are acquired through the appressorium and images presented as maximum intensity projections (as shown in Fig.1B), it's clear that the structure is a little more complicated than this, and the central pore becomes less apparent. To illustrate this fact, and hopefully make the septin ring in Figure 1B more interpretable, we have added a new panel (C) which shows a fully formed septin ring, and corresponding Tea1 signal, in a single Z plane and as a maximum intensity projection. Figure 1B shows frames from a time-series in which a single cell was imaged in ~60 Z planes, in two colours, every 30 minutes for a period of 9.5 hours. These imaging parameters were optimized to capture important cellular transitions. Inevitably, there is some photobleaching and loss of signal, which is evident in the Sep5-RFP channel at the 9.5 h timepoint. There is no intended "misrepresentation" of the data, and we strive to be as transparent in our data presentation and quantitation as possible. Showing a single cell over an entire time series, rather than selecting representative images from multiple different cells as is typical for the *Magnaporthe* field, is an important part of that. We hope that, provided with this additional panel (C), the septin ring in panel B (9.5 h) is easier to visualize and interpret.

5- Figure 2A. In the selected image, the Tea1-GFP signal is comparable between the wildtype and

Δsep3 samples. The only noticeable difference between the two genotypes is the size of the Tea1-GFP disc at 6 and 14 hpi. This image does not represent the claims made in the text. Either change the claims in the text or find an image that illustrates your claim. This problem may be due to the overlapping colors in the wildtype image. Separating the colors in the wildtype panel may reveal the appearance of the Tea1-GFP for proper comparison with the Δsep3.

We thank the reviewer for this critique, but disagree with their interpretation of the microscopy data, and point to the careful quantitation of the terminal phenotype (Fig.2B) which supports our claims within the text. Firstly, as requested by the reviewer in the first round of revisions, we have annotated the aberrant Tea1 patches seen only in the Δsep3 mutants (white arrow). Further, at the 6.0 timepoint, the Tea1-GFP single is interwoven with the septin structure in the wildtype appressorium. This is not the case in the corresponding Δsep3 mutant panel. There is no higher order septin structure, and there are no Tea1-GFP filaments. The Tea1-GFP signal is restricted to some ring-like puncta. Again, we have tried to be as transparent as possible with our data, by including the remodeling of single cells over time, and then carefully quantifying the terminal phenotypes to provide more robust support for our claims. We hope that the inclusion of Fig.1C, might also help in the interpretation of Figure 2A and B.

6- Figure 2I and J show that the fluorescence intensity of Lifeact-RFP and Tea1-GFP are lower in Δtea4 samples. However, the image shown in Figure 2G shows comparable fluorescence intensity for both markers. I assume that the Δtea4 image in 2G was processed to enhance the signal. If so, please state this difference in processing in the figure legend. If not, where does this quantification come from? How can these two images be so similar while the quantification of fluorescence intensity show such a decrease in signal in the mutant?
We apologize for any confusion. The images in Figure 2G are scaled to enable comparison of structural organization (2H), rather than fluorescence intensities, which are quantified in other panels (2I-J). We have added a statement to the figure legend “Brightness and contrast of representative images are not scaled equally” to clarify this.

7- Figure 3H and 3I are confusing and don't add anything to the information shown in Figure 3G. I suggest deleting these panels to avoid confusion.
We agree and have removed them.

8- Figure 3J does not illustrate the claim made in the text where the figure panel is mentioned. The sentence in question is "Unlike in non-branch associated septa, where cortical Tea1-GFP persists indefinitely (Fig.3B-F,J)." Figure 3J does not show indefinite Tea1-GFP maintenance. This figure panel should be deleted.
While we have removed these images, they did actually support the claims in the text, but we fully understand why the reviewer disagreed. In this older hypha, rather than the septin rings undergoing full contraction, they remained as smaller rings. We don’t know the biological significance of this but speculate that septin-mediated septum remodeling might be important for intercellular communication.

Possible mistake in a sentence:

9- Please verify the following sentence: "Thus, the redistribution of Tea1-containing polarity nodes to the periphery of cortical septin discs proceeds septin disc-to-ring remodeling, and these nodes spatiotemporally colocalize with sites of F-actin nucleation."
Did you mean "precedes" as in "it happens before"?
We thank the reviewer for catching this and have edited the text.

Errors related to the movies.

10- This manuscript file contains 4 movie files yet only 3 are referenced in the text.

We apologize for this error. In this revision, we have had to remove one of the movies to accommodate the model as a supplementary figure, so there are only 3 movies now, all of which are referenced in the text.

11- The movies are called out of order starting with Movie S1, S3 and S2.

We apologize for this and have corrected.

12- "Interestingly, Lifeact-RFP-labelled F-actin failed to accumulate within the appressoria of Δtea4 mutants to the same extent that it did in wildtype cells during their maturation (Fig.2E), and furthermore, did not localize to conspicuous contractile F-actin rings (Movie S3)". I think you mean Movie S4 as it's the one movie that illustrates these events.

Apologies, this has been corrected.

13- Movies S3 and S4 are too fast to appreciate the events they illustrate. Please slow them down.

We agree and have reduced the frame rate of these from 10 to 5 fps.

Minor suggestions:
14- "Following the maturation of wildtype appressoria, Lifeact-RFP-labelled F-actin filaments decorated Tea1-GFP- labelled cortical rings and appeared tightly interwoven with these structures". F-actin filaments is redundant. Choose "Lifeact-RFP-labelled F-actin" or "Lifeact-RFP-labelled actin filaments".

We agree with the reviewer and have edited the text accordingly.

15- You have valuable timing data from your movies. If you found a way to label one event as "time 0", you could add the timing data to your diagrams. This timing information would provide valuable information for the field.

Many thanks for the suggestion. We intend to carefully address this in a future review article.

All the best,

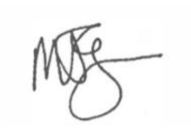

Martin Egan, PhD
Associate Professor
Department of Entomology and Plant Pathology
Cell & Molecular Biology
University of Arkansas
Fayetteville, AR 72701

RE: Manuscript #E23-06-0214RR
TITLE: "The cell-end protein Tea4 spatially regulates hyphal branch initiation and appressorium remodeling in the blast fungus Magnaporthe oryzae"

Dear Martin:

I am pleased to accept your manuscript for publication in Molecular Biology of the Cell. I appreciate your work addressing all of the comments from the reviewers and think the manuscript became more clear in the process. Thanks for sending this work to MBoC!

Sincerely,
Amy Gladfelter
Monitoring Editor
Molecular Biology of the Cell

Dear Dr. Egan:

Congratulations on the acceptance of your manuscript! Thank you for choosing to publish your work in Molecular Biology of the Cell (MBoC).

Within 10 days, an unedited PDF of your manuscript will be published on MBoC in Press, an early release version of the journal. The date your manuscript appears on this site is the official publication date.

Your copyedited and typeset manuscript will be scheduled for publication in the next available issue of MBoC. Our production team will notify you when the page proofs of your paper are ready for your review.

MBoC offers the option to publish your paper with immediate open access. Open access can increase the discoverability and usability of your research. If you would like to publish your paper with immediate open access but did not select this option during initial submission, please contact the MBoC Editorial Office (mbc@ascb.org).

We invite you to submit images related to your accepted manuscript to be considered for the journal cover. Please contact mboc@ascb.org to learn how to submit candidate cover images.

Authors of Articles and Brief Communications are also encouraged to create a short video abstract to accompany their article when it is published. These video abstracts, known as Science Sketches, are up to 2 minutes long and will be published on YouTube and then embedded in the article abstract. Science Sketch Editors on the MBoC Editorial Board will provide guidance as you prepare your video. Information about how to prepare and submit a video abstract is available at www.molbiolcell.org/science-sketches. Please contact mboc@ascb.org if you are interested in creating a Science Sketch.

We look forward to publishing your paper in MBoC.
Sign up for MBoC's new content alerts here https://tinyurl.com/3bcsyk3e

Sincerely,

Eric Baker
Journal Production Manager
MBoC Editorial Office
mbc@ascb.org
